# The Association between the Level of Advanced Glycation End Products and Objective Skin Quality Parameters

**DOI:** 10.3390/life13020256

**Published:** 2023-01-17

**Authors:** Dinko Martinovic, Daria Tokic, Mislav Usljebrka, Slaven Lupi-Ferandin, Livia Cigic, Lucija Vanjaka Rogosic, Sasa Ercegovic, Mirko Kontic, Marko Kumrić, Doris Rusic, Marino Vilovic, Mateo Leskur, Josko Bozic

**Affiliations:** 1 Department of Maxillofacial Surgery, University Hospital of Split, 21000 Split, Croatia; 2 Department of Anesthesiology and Intensive Care, University Hospital of Split, 21000 Split, Croatia; 3 Department of Dermatovenerology, University of Split School of Medicine, 21000 Split, Croatia; 4 Private Dermatovenerology Clinic, 21000 Split, Croatia; 5 Department of Otorhinolaryngology, University Hospital of Split, 21000 Split, Croatia; 6 Department of Pathophysiology, University of Split School of Medicine, 21000 Split, Croatia; 7 Department of Pharmacy, University of Split School of Medicine, 21000 Split, Croatia

**Keywords:** skin, advanced glycation end products, Melanin, TEWL, skin barrier, physiology

## Abstract

Advanced glycation end products (AGEs) represent an endogenously produced or exogenously derived group of compounds derived from nonenzymatic glycation. Recent experimental studies are suggesting that AGEs could play an important role in the skin’s quality and its aging process. Hence, the aim of this study was to clinically evaluate the AGEs and skin quality parameters across different age groups in the general population. The study included 237 participants. Melanin, erythema, hydration, friction and transepidermal water loss (TEWL) were evaluated using noninvasive probes, while AGEs were evaluated using a skin autofluorescence reader. There was a significant positive correlation between AGEs and the amount of melanin (*p* < 0.001), erythema (*p* < 0.001) and TEWL (*p* < 0.001), while there was a significant negative correlation between AGEs and hydration (*p* < 0.001) and friction (*p* < 0.001). After dividing the sample into three groups depending on their age, in all three groups, there was a significant positive correlation between AGEs and the melanin count (*p* < 0.001) and TEWL (*p* < 0.001), while there was a significant negative correlation between AGEs and skin hydration (*p* < 0.001). Multiple linear regression analysis showed that the level of AGEs as a dependent variable retained a significant association with age (*p* < 0.001), melanin (*p* < 0.001), erythema (*p* = 0.005) and TEWL (*p* < 0.001) as positive predictors. Moreover, AGEs retained a significant association with skin hydration (*p* < 0.001) and friction (*p* = 0.017) as negative predictors. These outcomes imply that AGEs could be linked with the complex physiology of the skin and its aging process.

## 1. Introduction

Skin aging is a complex, multifactorial process that could be described as a progressive accumulation of damage that leads to dysfunction on both the cellular and tissue levels [1,2]. Mutually genetic, endogenous and environmental factors have an impact on this process [3]. Throughout the aging process, there is a reduction in the number of fibroblasts that synthesize collagen and the number of vessels that supply the dermis, which consequently leads to an increase in laxity and therefore the formation of wrinkles [4,5]. Moreover, the epidermis becomes significantly thinner with higher age, while collagen and elastin, two of the main constituents of dermal tissue, also undergo significant alteration [6]. In intrinsically aged skin, the quantity of dermal collagen diminishes while elastin accumulates structural abnormalities [7]. On the other hand, extrinsically aged skin, or photoaged skin, is characterized by the hyperplasia of the elastic tissue and thicker epidermis. Some studies have shown that the amount of dermal collagen present in the sun-protected skin of people aged over 80 years deteriorates by 75% compared to the skin of young adults [8]. The skin is also accompanied by sebaceous, eccrine and apocrine glands, which have an important role in the thermoregulation process and also in re-epithelization after injuries. Although the number of sebaceous glands remains relatively the same throughout the lifetime, their metabolic activity changes by intrinsic and extrinsic factors, as well as the aging process [9].

Advanced glycation end products (AGEs) represent an endogenously produced or exogenously derived group of compounds derived from the nonenzymatic glycation of sugars, proteins, lipids or nucleic acids through the Maillard reaction [10]. The Maillard reaction is a nonenzymatic chemical reaction between reducing sugars and amino acids. The products of this reaction are described as AGEs and were initially recognized in cooked foods. AGEs can be produced endogenously as well as exogenously, ingested via food or influenced by other environmental factors [11]. Given that glycation is a continuous process during aging and that the glycated compounds disintegrate at a very slow rate, AGEs accumulate with rising age. This is further augmented by hyperglycemia, hyperlipidemia, oxidative stress and inflammation [12]. Through the multiligand receptor for advanced glycation end products (RAGE) on the vascular cells, AGEs play a key role in the inflammatory process [13]. It is well established that higher levels of AGEs are linked to diseases such as diabetes, chronic kidney disease, nonalcoholic fatty liver disease, obesity and cardiovascular diseases [14,15,16,17,18,19].

While AGEs were mostly investigated through cardiovascular diseases and diabetes, in recent years there have been a lot of studies that are linking the level of AGEs with the quality of the skin and discussing the possibility of AGEs playing an important role in skin aging [20,21,22]. The easy accessibility of skin offers a chance of studying AGEs via minimal invasive or even noninvasive methods due to their characteristic autoflorescent properties [20]. Studies have found that young individuals show little accumulation of AGEs in sun-protected skin, while on the other hand, they have larger amounts of AGEs accumulated in sun-exposed parts of skin [23,24]. Other extrinsic factors, including smoking and diet, also contribute to the formation of AGEs that get deposited in the skin [25]. Moreover, it was shown that the major targets for glycation are extracellular matrix proteins such as collagen, elastin and fibronectin. The glycation of collagen disrupts its function in many ways leading to decreased elasticity, therefore, making it more prone to mechanical stimuli [9]. Furthermore, it was shown that AGEs reduce proliferation and facilitate apoptosis of dermal fibroblasts, a process that is partially RAGE-dependent and associated with several pathophysiological mechanisms [26].

Hence, the aim of our study was to investigate the level of AGEs and the objective skin quality parameters across different age groups. Furthermore, the goal was to evaluate the possible connection between the amount of melanin, erythema, friction, hydration and transepidermal water loss (TEWL) with the level of AGEs.

## 2. Materials and Methods

### 2.1. Study Design and Ethical Considerations

This cross-sectional observational study was conducted at the Department of Maxillofacial Surgery, University of Split School of Medicine during the period from February 2022 to June 2022.

All subjects were informed about the purpose, aims and procedures of the study before its onset. Moreover, all of the included subjects signed a written informed consent to participate in this study. The Ethics Committee of the University of Split School of Medicine approved the study (No: 003-08/22-03/0003) and it was performed in accordance with the latest Declaration of Helsinki.

### 2.2. Subjects

The study included volunteers who were recruited through healthcare workers, acquaintances, students and friends. The inclusion criteria were: age between 18 and 80 years and Fitzpatrick skin types I–III. The Fitzpatrick classification system categorizes skin based on the tan in six types:

I: those who have pale skin and freckles, always sunburn and do not tan at all;

II: those who have light-colored skin, sunburn easily and tan with difficulty;

III: those who have olive/golden honey skin, sunburn mildly, have immediate pigment darkening and tan uniformly;

IV: those who have moderate brown skin, burn minimally and always tan well;

V: those who have dark brown skin, rarely burn and tan easily;

VI: those who have deeply pigmented dark brown to darkest brown skin that never burns.

The exclusion criteria were: scars, tattoos or other skin marks on the volar side of the forearm of the dominant hand; active malignant disease; diabetes mellitus; cardiovascular disease; hepatic and renal disease; chronic and acute skin disease; skin xerosis; smoking; and excessive alcohol consumption. The medical records of all potential subjects were checked prior to their inclusion in the study. There were 281 subjects screened for inclusion; however, 44 subjects did not meet the aforementioned criteria. Hence, the final number of included subjects was 237.

### 2.3. Objective Skin Assessment

The skin quality assessment was conducted by the same experienced investigator using the MP6 skin quality evaluation instrument (Courage + Khazaka GmbH, Cologne, Germany). This instrument evaluates several skin qualities with noninvasive probes [27,28,29]. Transepidermal water loss (TEWL) as an objective indication of skin barrier function and was estimated using the Tewameter^®^ TM 300. Skin hydration was estimated using the Corneometer^®^ CM 825. The amount of skin melanin and erythema were estimated using the Mexameter^®^ MX 18. Lastly, skin friction, as a biophysical property that indicates the resistance against the movement of objects on the skin, was estimated using the Frictiometer^®^ FR 700. Prior to the study onset, all of the probes were calibrated according to the manufacturer’s instructions.

All of the participants were subjected to measurements in a room that had carefully monitored air conditions. Air humidity was retained at 40–55% using a Philips 3000i air humidifier (Koninklijke Philips N.V., Amsterdam, Netherlands), while the room temperature was kept at 20–22 °C using the inbuilt air conditioners. The participants were first seated in the room for 20 min to acclimatize the skin to the aforementioned conditions. Participants were instructed to take a shower on the morning of the measurement day and to strictly avoid using any make-up, skin creams or any other skin preparations. All measurements were conducted on the forearm of the dominant hand. The probes were held at a right angle to the skin and gently applied to the skin for optimal contact; all measurements were performed three times after which the mean value was calculated. After every participant, the probes were disinfected and prepared for the next subject.

Additionally, to test the possible intraobserver variability, several of the participants underwent the objective evaluation using the probes on three (3) different days. There was no statistically significant difference between their results.

### 2.4. AGEs Measurement

The levels of AGEs were measured using the skin autofluorescence (SAF), which was estimated by the same experienced investigator using the AGE-Reader SU (DiagnOptics Technologies BV, Groningen, The Netherlands) [10,30]. SAF was calculated as the average light intensity in the 420–600 nm range divided by the average light intensity in the 300–420 nm range, and the result was expressed in arbitrary units (AU). Measurements of SAF were performed on the same location on the dominant forearm as the prior skin assessment. The dominant forearm of patients was positioned on the device for three consecutive measurements, and the mean of three measurements was calculated to achieve an accurate AGEs value.

### 2.5. Statistical Analyses and Sample Size Calculation

All statistical analyses were conducted using the statistical software MedCalc for Windows^®^ (version 20.110, MedCalc Software, Ostend, Belgium), while all graphical figures were created using the SigmaPlot for Windows^®^ (version 14.0, Systat Software Inc, San Jose, CA, USA). The normality of distribution was evaluated using the Kolmogorov–Smirnov test. All qualitative variables were presented as a whole number (percentage). All normally distributed continuous quantitative variables were presented as mean ± standard deviation, while all non-normally distributed continuous quantitative variables and all noncontinuous quantitative variables were presented as median (interquartile range). Using the statistical software, three tertiles, depending on the age of the subjects, were created: younger age group (<33 years, N = 80), middle age group (33–56 years, N = 80) and older age group (>56 years, N = 77). The comparison between the three groups was performed using a one-way analysis of variance (ANOVA) with post hoc Scheffe’s test or using the Kruskal–Wallis test with post hoc Dunn’s test. A correlation was calculated using Pearson’s correlation coefficient or Spearman’s correlation coefficient. Lastly, multiple linear regression analyses were performed to determine significant independent predictors for the AGEs levels. From these analyses, we reported the *p*-values with unstandardized β-coefficients, standard errors and *t*-values. The level of statistical significance was set at a *p*-value < 0.05.

Since the correlation coefficients were among the main outcomes of this study, with a type I error of 0.05 and a power of 90%, to achieve the moderate correlation coefficient of 0.5, the required sample size was 37 participants per group.

## 3. Results

There were 142 (59.9%) male subjects and 95 (40.1%) female subjects in the study sample. The median age was 41.0 (22.0–62.0) years and the median BMI was 24.5 (22.2–26.4) kg/m^2^ (Table 1). The older age group had a significantly higher body mass (*p* < 0.001), body height (*p* = 0.009) and BMI (*p* < 0.001) compared to the middle and younger age groups (Table 1). Moreover, the older age group had a significantly higher melanin count (*p* = 0.010) and hydration (*p* < 0.001) than the other two groups, while friction was significantly lower than the other two groups (*p* < 0.001) (Table 1). Additionally, the younger age group had significantly lower erythema compared to the older age group (*p* = 0.028) (Table 1).

**Figure 1:**
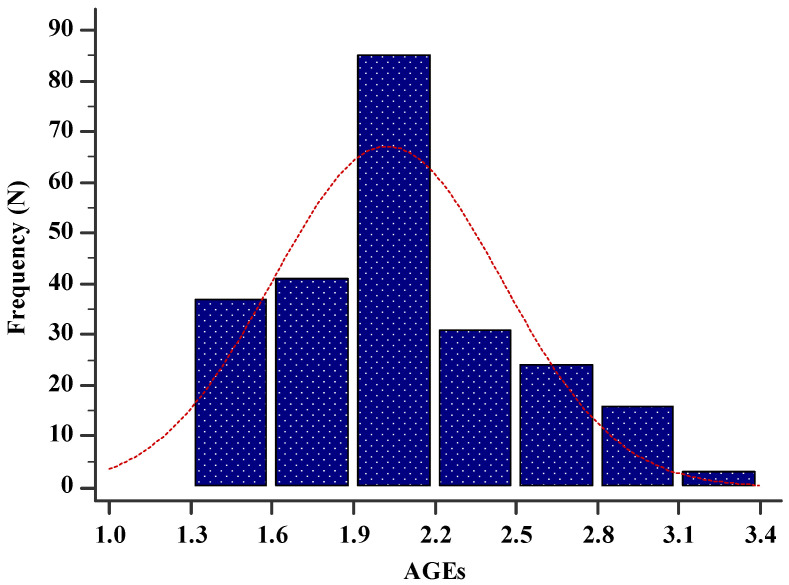
Histogram showing the distribution of AGEs in the study sample (N = 237). Abbreviations: AGEs—advanced glycation end products.

**Figure 2:**
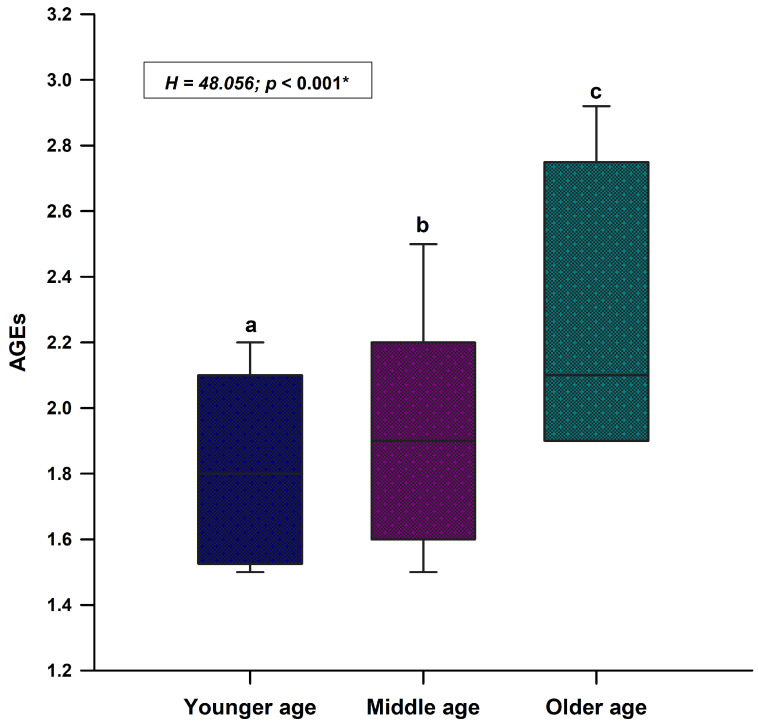
Comparison of AGEs values between the younger age group (<33 years; N = 80), the middle age group (33–56 years; N = 80) and the older age group (>56 years; N = 77). Abbreviations: AGEs—advanced glycation end products. * Kruskal–Wallis test with post hoc Dunn’s test. p < 0.05—a vs. b; a vs. c; b vs. c.

The mean value of AGEs in the study sample was 1.9 (1.7–2.2) (Figure 1). There was a statistically significant difference regarding the value of AGEs between all three different age groups (H = 48.05; *p* < 0.001) (Figure 2). The older age group had the highest AGEs values while the younger age group had the lowest AGEs values (Figure 2).

**Figure 3:**
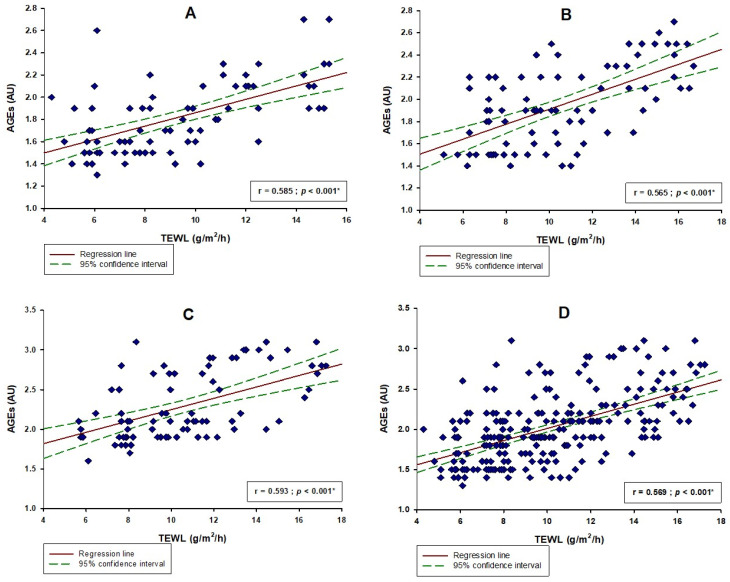
Correlation between AGEs and TEWL in (**A**) the younger age group (<33 years; N = 80), (**B**) the middle age group (33–56 years; N = 80), (**C**) the older age group (>56 years; N = 77) and (**D**) the whole study sample (N = 237). Abbreviations: AGEs—advanced glycation end products; TEWL—transepidermal water loss. * Spearman’s correlation.

There was a statistically significant positive correlation between the level of AGEs and the TEWL in the whole study sample (r = 0.585, *p* < 0.001) (Figure 3D). There was a statistically significant positive correlation between the values of AGEs with melanin count (r = 0.488; *p* < 0.001), erythema values (r = 0.237; *p* < 0.001) and TEWL (r = 0.585; *p* < 0.001) in the whole study sample (Table 2). Additionally, there was a significant negative correlation between the values of AGEs with hydration (r = −0.501; *p* < 0.001) and friction (r = −0.307; *p* < 0.001) (Table 2).

Furthermore, melanin count showed a significant positive correlation with AGEs in the younger age group (r = 0.482; *p* < 0.001), middle age group (r = 0.499; *p* < 0.001) and the older age group (r = 0.491; *p* < 0.001) (Table 2). Hydration also showed a significant negative correlation with AGEs in the younger age group (r = −0.492; *p* < 0.001), middle age group (r = −0.478; *p* < 0.001) and the older age group (r = −0.522; *p* < 0.001) (Table 2). Lastly, TEWL showed a significant positive correlation with AGEs in the younger age group (r = 0.585; *p* < 0.001), middle age group (r = 0.565; *p* < 0.001) and the older age group (r = 0.593; *p* < 0.001) (Figure 3).

Multiple linear regression analysis showed that the level of AGEs as a dependent variable retained a significant association with age (β ± SE, 0.006 ± 0.001, *p* < 0.001), melanin (0.002 ± 0.0005, *p* < 0.001), erythema (0.001 ± 0.0002, *p* = 0.005) and TEWL (0.027 ± 0.006, *p* < 0.001) as positive predictors. Moreover, it retained a significant association with hydration (−0.007 ± 0.002, *p* < 0.001) and friction (−0.001 ± 0.0001, *p* = 0.017) as negative predictors (Table 3).

## 4. Discussion

The outcomes of this cross-sectional study have shown that in the whole study sample, there was a significant positive correlation between the level of AGEs with the amount of melanin, erythema and TEWL, while there was also a significant negative correlation between AGEs with hydration and friction. However, since age is a well-established factor with a major influence on both the level of AGEs and skin quality, subanalyses were conducted on different age groups to diminish this age-related impact on the implied association between skin quality and AGEs. In the younger age group, the same significant positive correlation was found regarding the level of AGEs with the melanin count and TEWL, while there was also a significant negative correlation between AGEs and skin hydration. Furthermore, these same correlations were found in the middle age and older age groups. However, there was no significant association between erythema and friction with the level of AGEs. Additionally, a multiple linear regression analysis was conducted and it showed that melanin, erythema and TEWL were independent significant positive predictors of the level of AGEs, while the amount of hydration and friction were significant negative predictors.

Accumulation of AGEs is deemed important in skin aging because it consequently facilitates oxidative stress and inflammation [31]. Pigmentation of the skin represents a barrier to damage caused by intrinsic and extrinsic factors such as UV exposure and different hormones and is considered one of the main signs of photoaging. The results of our study are in line with a recent study that found that AGEs promote melanogenesis through their receptors [32]. They confirmed the existence of RAGEs in melanocytes and they showed that keratinocytes influenced by UV irradiation induce the production of AGEs, which subsequently stimulates the production of melanin through RAGE expression. Another recent study that observed sun-exposed skin in ex vivo cultured skin and mouse models showed that AGEs were positively correlated with epidermal melanin levels [33]. Moreover, they found that AGEs potentially activated NLRP3 inflammasome and induced IL-18 production in fibroblasts, all of which were mediated through RAGE. All of the aforementioned outcomes are suggesting that AGEs could be playing a pivotal role in photoaging.

Furthermore, our study also showed that AGEs and TEWL have a significant positive correlation. TEWL is a crucial component when considering the skin barrier as it assesses the stratum corneum function [34,35]. Higher TEWL signifies that the permeability of the skin is increased, which means that its integrity is disrupted and, consequently, skin hydration, another important skin factor, is also disturbed [36]. According to our knowledge, this is the first study investigating the association between TEWL and AGEs. However, our outcomes are partially in line with other similar studies investigating AGEs and their effects on the skin [37,38,39]. A previous study has shown that AGEs reduce the expression of ceramide synthase (CERS3) which, consequently, results in lower amounts of ceramide and cholesterol in the epidermis, therefore, destroying the skin barrier [37]. Reduced epidermal lipid synthesis causes the reduction in skin lamina and delays the self-repair of the skin. Glycation also affects the function of epidermal structural proteins such as filaggrin and transglutaminase-1, thereby causing further damage to the skin barrier [39]. Since skin sebum is an important link in the skin barrier function, all of these studies, including ours, are implying that AGEs are potentially destroying the skin barrier and lowering its protective function. However, it is important to note that a recent systematic review showed that TEWL is lower in individuals aged over 65 years [40]. While we did not find such a difference between younger and older subjects, this could be explained by the fact that the cut-off value for our older age group was >56 years. Moreover, our region has had a large number of sun hours throughout the years and several studies have found that sun exposure can have an impact on TEWL [41]. Nevertheless, TEWL needs to be further explored regarding its association with the aging process.

Due to the close relationship between TEWL and hydration, it is important to note our outcome of a significant negative correlation between AGEs and skin hydration. Water is essential for skin function and several crucial mechanisms depend on the water gradient in the skin, one of them is the proteolysis of filaggrin by keratinocytes, and as abovementioned, AGEs interfere with the function of keratinocytes [42]. Considering this, it appears that not only do AGEs directly disrupt the skin as a barrier by obstructing the physiological functions, but they also affect it indirectly by increasing TEWL, which causes further and severe damage to the skin on multiple levels.

Lastly, we found a significant negative correlation between skin friction and AGEs. Friction is a biomechanical property of the skin and it indicates its resistance to the movement of objects. Previous studies established that skin friction is positively associated with skin hydration; however, it is still unclear how the aging process influences this parameter [43]. Several studies reported that there are no significant differences between younger and older subjects, while on the other hand, several studies reported a significant difference [44,45,46]. Our outcome, which implies an association with the level of AGEs, could be elaborated by its association with the stratum corneum hydration, which was also found in earlier biomechanical studies [44]. Since the results of our study are implying that AGEs are possibly influencing a reduction in skin hydration due to the persistently disrupted barrier, which is also demonstrated with a higher TEWL, perhaps AGEs are also indirectly impacting skin friction through this association.

There are several limitations to this study. First of all, its cross-sectional design diminishes the possibility of making any causal conclusions. Dietary habits play a role in glycation and, consequently, they have an impact on the level of AGEs; however, it was impossible to eliminate all of the confounding effects. The sample size is relatively small and the study was conducted in only one center/region. Additionally, our region has a high number of sun hours throughout the year; even though the study was conducted before the summer, the continuous solar exposure may have interfered with our results. Furthermore, the noninvasive methods that were used for evaluating skin parameters could potentially have been affected by inter and intraobserver variability. However, we believe that this was diminished by using only one investigator and conducting three measurements, from which the mean value was calculated. Lastly, we did not measure skin sebum due to its particularly low values on the volar side of the forearm, which would cause statistical analyses and any interpretation of the results to be unclear.

## 5. Conclusions

In conclusion, this study found that there is a significant association between the quality of the skin and the level of AGEs. Melanin count, erythema and TEWL exhibited a significant positive correlation, while hydration and friction have shown a significant negative correlation with AGEs. All of these results are implying that AGEs could be playing an important role in skin physiology with possible significant impacts on the aging process. Furthermore, these outcomes are in alignment with other recent studies concerning the role of AGEs in the skin, and it furtherly widens the future perspective for investigating AGEs and how they intervene with skin physiology. However, as aforementioned, AGEs are only a product of both our lifestyle and genetic predisposition and as such, they are possibly just a link in the complex network that influences skin aging. Nevertheless, these results should be furtherly addressed in a larger longitudinal multicentric study.

## Figures and Tables

**Table 1:** Anthropometric characteristics and objective skin quality parameters of the study sample.

Parameter	Study Sample (N = 237)	Younger Age (<33 Years) N = 80	Middle Age (33–56 Years) N = 80	Older Age (>56 Years) N = 77	p ^†^
Male gender (N, %)	142 (59.9)	48 (60)	46 (57.5)	48 (62.3)	0.825
Age (years)	41.0 (22.0–62.0)	21.0 (19.0–22.0)	41.5 (37.0–48.0)	67.0 (62.0–74.0)	<0.001 ^abc^
Body height (cm)	178.0 (170.0–185.0)	177.0 (170.0–185.0)	174.0 (167.0–181.5)	181.0 (175.0–186.0)	0.009^c^
Body mass (kg)	78.2 ± 8.9	77.2 ± 15.2	76.7 ± 15.5	88.4 ± 16.6	<0.001 ^bc^
BMI (kg/m^2^)	24.5 (22.2–26.4)	24.5 (22.3–26.3)	24.7 (23.0–27.4)	27.4 (25.4–29.0)	<0.001 ^bc^
Melanin (AU)	99.6 (69.1–133.8)	94.2 (66.0–127.7)	90.8 (65.4–124.4)	112.0 (78.2–145.1)	0.010 ^bc^
Erythema (AU)	256.2 ± 75.4	244.1 ± 75.4	250.4 ± 78.1	274.6 ± 69.9	0.028 ^b^
Hydration (AU)	48.8 (41.0–60.3)	50.6 (44.2–62.6)	50.9 (41.8–63.4)	45.2 (37.1–53.9)	<0.001 ^bc^
Friction (AU)	249.0 (145.0–378.5)	273.4 (189.2–462.2)	297.8 (181.1–465.2)	190.1 (125.4–301.8)	<0.001 ^bc^
TEWL (g/m^2^/h)	9.7 (7.6–12.4)	9.3 (6.9–12.2)	9.7 (7.5–12.7)	9.9 (7.9–12.8)	0.165

Data is presented as whole number (percentage), mean ± standard deviation or median (interquartile range). Abbreviations: BMI—body mass index; TEWL—transepidermal water loss. ^†^ chi-square test or one-way analysis of variance (ANOVA) with post hoc Scheffe’s test, or Kruskal–Wallis test with post hoc Dunn’s test. post hoc *p*-value < 0.05: ^a^ younger vs. middle; ^b^ younger vs. older; ^c^ middle vs. older.

**Table 2:** Correlation of AGEs with the skin quality parameters in the whole study sample and the different age groups.

Parameter	Study Sample N = 237 r (*p*)	Younger Age (< 33 Years) N = 80 r (*p*)	Middle Age (33–56 Years) N = 80 r (*p*)	Older Age (>56 Years) N = 77 r (*p*)
Melanin (AU)	0.488 (<0.001) ^‡^	0.482 (<0.001) ^‡^	0.499 (<0.001) ^‡^	0.491 (<0.001) ^‡^
Erythema (AU)	0.237 (<0.001) †	0.162 (0.150) †	0.180 (0.102) †	0.193 (0.092) ^†^
Hydration (AU)	−0.501 (<0.001) ^‡^	−0.492 (<0.001) ^‡^	−0.478 (<0.001) ^‡^	−0.522 (<0.001) ^‡^
Friction (AU)	−0.307 (<0.001) ^‡^	−0.191 (0.089) ^‡^	−0.192 (0.087) ^‡^	−0.224 (0.051) ^‡^

Abbreviations: AGEs—advanced glycation end products; BMI—body mass index; TEWL—transepidermal water loss. ^†^ Pearson’s correlation ^‡^ Spearman’s correlation.

**Table 3:** Multiple linear regression analysis of independent predictors for AGEs values in the whole study sample (N = 237).

Parameter	β ^†^	SE ^‡^	t	*p*
Age	0.006	0.001	5.879	<0.001
BMI	−0.012	0.006	−1.811	0.071
Melanin	0.002	0.0005	4.834	<0.001
Erythema	0.001	0.0002	2.802	0.005
Hydration	−0.007	0.002	−3.901	<0.001
Friction	−0.001	0.0001	−2.394	0.017
TEWL	0.027	0.006	4.084	<0.001

Abbreviations: AGEs—advanced glycation end products; BMI—body mass index; TEWL—transepidermal water loss. ^†^ Unstandardized coefficient β. ^‡^ Standard error.

## Data Availability

All data sets are available upon request to the corresponding author.
